# Digital technology innovation, supply chain resilience and enterprise performance-The case of listed automotive parts manufacturing companies

**DOI:** 10.1371/journal.pone.0313929

**Published:** 2025-01-09

**Authors:** Xiangbing Chen, Chen Sun, Fang Wang

**Affiliations:** Wuhan University of Science and Technology, Hubei Province, China; Abu Dhabi University, UNITED ARAB EMIRATES

## Abstract

Digital technology innovation (DTI) is the core driving force for the development of the digital economy. This paper brings digital technology innovation and the supply chain of auto parts manufacturing under the same framework. This paper uses Stata 18 to empirically analyze the panel data of 130 A-share auto parts listed companies in Shanghai and Shenzhen from 2010 to 2022. The digital technology innovation indicator is divided into three levels: substantial digital technology innovation (SDTI), non-substantial digital technology innovation (NDTI), overall digital technology innovation (ODTI). To explore its impact mechanism on enterprise performance. The empirical results show that: (1) Digital technological innovation (DTI) has a positive and significant impact on enterprise performance, and supply chain resilience plays a mediating role in the relationship between digital technology innovation and enterprise performance, and R&D investment (RDI) positively moderates the role of supply chain resilience in promoting enterprise performance. (2) Heterogeneity analysis showed that the impact of supply chain resilience on enterprise performance was more significant in small and medium-sized enterprises. There are significant differences between different groups of business ownership. In economically underdeveloped regions, the effect of digital technology innovation on enterprise performance is more significant. This paper complements the perspective of supply chain to study the relationship between digital technology innovation and enterprise performance, expands the existing research, and its heterogeneity analysis provides new insights for understanding China’s auto parts manufacturing industry. This provides a basis for strengthening digital technology innovation and promoting the sustainable development of the auto parts industry.

## 1. Introduction

With the deepening and development of the digital revolution, many fields have undergone significant changes. Digital technologies based on artificial intelligence, big data, cloud computing, and blockchain have become the key forces driving this change, and the world has entered the era of big data [1]. Under the new development pattern with data at the core, competition in the industry has gradually evolved into a competition for data acquisition capabilities and the ability of enterprises to transform data resources into productivity [2]. In 2023, The scale of China’s digital economy reached 53.9 trillion yuan, an increase of 3.7 trillion yuan from the previous year, accounting for 42.8% of GDP. The digital economy grew by 7.39% year-on-year in nominal terms [3].

Industry 4.0 revolutionizes the operation model of global production and supply chain networks by automating traditional manufacturing processes, such as the introduction of cyber-physical systems (CPS) and the application of Internet of Things (IoT) and big data technologies. The application of Industry 4.0 technology integrates all sub-systems into a single system to form a self-monitoring mechanism [4]. If the manufacturing industry wants to optimize production methods to cope with the increasingly complex industry landscape, it must adopt advanced technological means to innovate production processes [5]. In the context of Industry 4.0, digital technologies can lead to the transformation of business models, which can improve production processes, improve the efficiency of equipment operation, making production management more accurate, at the same time, help to reduce resource costs [6], the reduced production costs can be used for the next round of investment in DTI activities [7].

The supply chain of automotive manufacturers are characterized by high degree of complexity and diversity on global scale [8]. Automakers in the United States, Europe, Japan and Korea are important bases for the global automotive industry, selling cars and auto parts not only in their home markets, but also around the world through a global distribution network [9]. Compared with developed countries, there is a certain gap between China’s automobile manufacturing industry and developed countries in terms of core technology and innovation capabilities, manufacturing and supply chain management, etc. In order to narrow these gaps, enterprises need to take the initiative to explore the application of digital technology, to promote DTI, in order to obtain a competitive advantage in the increasingly changing market, to achieve sustainable development [10].

COVID-19 has brought the topic of supply chain disruption to the attention of a wide range of scholars, and the lack of raw materials such as electronic components, chips, and semiconductors has had a huge impact on the automotive industry [6]. COVID-19 has prompted companies to restructure their supply chains in a demand-driven manner to improve SCR and reduce shortages. Digital technologies empower the manufacturing industry to transform the structure of the supply chain from a centralized production model to a distributed model [11]. This will shorten the supply chain to a certain extent, and the pressure from the technological innovation of core enterprises will enhance the innovation momentum of upstream and downstream enterprises in the supply chain [12]. At the same time, it promotes collaborative innovation between upstream and downstream enterprises in the supply chain, and enhances the competitiveness of the entire supply chain.

The existing literature mainly focuses on the impact of DTI on performance, including innovation performance and Internationalization Performance [7, 10, 13]. Obviously, this is an important question. On the one hand, due to the highly complex characteristics of the supply chain of the auto parts manufacturing industry and the unpredictable new market pattern, the importance of supply chain management has become more and more prominent, but the existing empirical research on DTI and enterprise performance lacks a supply chain perspective, what is the potential mechanism of the supply chain? On the other hand, due to the differences in China’s geographical environment and resource endowment, will there be regional differences in the mechanism of DTI on enterprise performance in different regions of China?

Therefore, the key of this study is to explore the impact mechanism of DTI on the performance of auto parts manufacturing enterprises in the era of digital economy. This study explores the differential impact of DTI on enterprises performance in the context of regional economic disparities in China, explore an interesting and meaningful question.

This study contributes to the existing literature in three aspects: First, it expands the existing research by exploring the relationship between DTI and enterprises performance from the perspective of the auto parts supply chain. Second, this paper considers the mediating variables of SCR in this relationship, and the moderating variable of R&D investment in this relationship. Finally, the heterogeneity of this relationship provides insight into China’s auto parts manufacturing industry due to the differences in enterprise size, business ownership and region.

The structure of this paper is as follows: Section 2“Literature review and hypotheses development” details the variable-based literature and hypothesis proposed. Section 3“Methodology”Covers data, variables, and models. Section 4 “Empirical analysis” presents the benchmark regression results, robustness tests, and heterogeneity analysis. Finally, Section 5 “Discussion and Conclusions” summarizes the findings, implications, limitations of the study, and suggestion for future research.

## 2. Literature review and hypotheses development

### 2.1 Digital technology innovation and enterprise performance

DTI has attracted the attention of many disciplines such as economics, social sciences, and education. Digital innovation is the transformation of traditional strategies and organizations through new combinations. Divide digital innovation into seven micro levels, which are: digital focus, digital innovation process, digital mindset, digital innovation network, digital technology capability [14], data management, and overcoming digital innovation resistance [15]. Enterprises aim to reorganize and allocate existing resources through the perception of digital innovation capabilities to maintain technological competitiveness [16]. Through digital technologies, changes are implemented to traditional management models and in the process, economic growth is accelerated and business model upgrades are achieved [17]. Based on the original data of electronics companies, analyzes the mechanism of technology innovation in promoting technology orientation and improving innovation performance, so as to help electronics companies strengthen management and control the corresponding production mechanism [18]. DTI integrates manufacturing and information systems (IS) to ensure data and process integration, improve customers’ advantages for mass customization, and the collaborative innovation of manufacturing, marketing and supply chain can bring performance benefits to enterprises [19].

DTI can prompt auto parts manufacturing enterprises can realize the automation and intelligence of the production process, which can improve the production efficiency, make data entry, processing and analysis more accurate and faster, further improve the quality and efficiency of production. Digital technology is the ability to search for and apply resources and knowledge to bring superior performance to a business [20]. Digital technology is an important cornerstone of the manufacturing industry to launch new products and services, DTI can improve the overall competitiveness of the manufacturing industry [21], It can improve the product design and R&D capability, enhance the market analysis and forecasting capability, and promote the performance of auto parts manufacturing enterprises. The opportunities brought about by DTI may be hindered by small and medium-sized enterprises, which are limited by resources and technical barriers, and often find it difficult to occupy a favorable position in the market competition [22]. In the process of sustainable practices, product-service can compensate for the disadvantages of this part of the company’s limited resources, so that they can innovate in the product cycle, thereby improving firm performance [23]. Based on this, we propose Hypothesis 1.

H1: DTI has a positive impact on enterprise performance.

### 2.2 Supply chain resilience mediates the relationship between digital technology innovation and enterprise performance

“Resilience” in physics is divided into fracture resilience and impact resilience. However, economic resilience is different from engineering resilience. SCR is the ability to recover from supply chain disruptions and maintain continuity of raw material, information, and cash flows [24]. Supply chain robustness is one of the dimensions of SCR and aims to address supply chain risks by reducing complex organizational structures, complicated processes, maintaining redundant resources, and developing more stable processes [25]. Tracing the concept of resilience, manufacturing resilience can also be categorized into fracture resilience and impact resilience. Fracture resilience is an inherent attribute of the manufacturing industry, which includes the volume and scale, condition endowment, stability and complexity of the system accumulated over a long period of time [26]. Impact resilience refers to the vulnerability of the supply chain and the external impact of the industry outlook. Existing studies generally agree that SCR has a positive impact on enterprise performance [27]. SCR can improve the competitiveness and financial performance of manufacturing enterprises, enabling enterprises to respond quickly to changes in the environment and actively adjust their strategies to prevent major disruptions in the supply chain [28].

SCR is a multi-dimensional, multi-layered structure that can also be categorized as Proactive Capability, Responsiveness, and Supply Chain Design Quality [29]. Firstly, supply chain proactive capability is the ability to identify, predict and defend against risks before adverse consequences occur. Supply chain research emphasizes different proactive capability such as diversity, effectiveness, adaptability and cohesion [28]. Secondly, supply chain responsiveness emphasizes the flexibility to combat risks, and the enhancement of responsiveness enables enterprise to maintain contact and collaboration with supply chain partners, quickly coordinate upstream and downstream supply chain resources, and ensure effective integration of supply chain business processes, which in turn improves enterprise performance [30]. Finally, supply chain design quality refers to the ability to proactively plan and design its network to anticipate subsequent supply chain disruptions and respond effectively to them, and to establish a diversified network of suppliers to diversify supply-generated risks, which increases the likelihood that an enterprise will quickly switch to other reliable partners in the event of a supply chain disruption, ultimately improving enterprise performance [31]. Big data and predictive analytics(BDPA) can have a positive impact on business performance by improving operational processes and integrating them into supply chain management(SCM) [32]. Scholars generally believe that digital technology can improve the operational efficiency of supply chains and improve the robustness and resilience of supply chains [33, 34]. In addition, from the resource-based view theory, it is believed that valuable, scarce, inimitable and irreplaceable resources are the key to improving enterprise performance and providing sustainable competitive advantage [35], while DTI can help such resources by further improving the transparency and visualization of the supply chain, reducing supply chain risks and increasing SCR, which in turn improves performance. Based on the above theory, Hypothesis 2 is proposed.

H2: SCR mediates the relationship between DTI and enterprise performance.

### 2.3 Enhancement of supplier’s RDI

R&D is mainly to improve the productivity of enterprises by reducing the production cost of existing products or improving the quality of products, In addition, conducting R&D in one firm/sector/country may have positive spillover effects on other firms/sectors/countries [36]. For Chinese manufacturing companies, the more productive they are, the more likely they are to choose R&D investment [37]. The study finds that information technology (ICT) R&D investment is driven by economic growth, and the private ICT R&D investment is more closely related to economic growth [38]. Enterprise investment in innovation is one of the dynamic capabilities to increase resilience. Innovation enhances an enterprise response to uncertain events by creating market value and enables enterprise to respond to fluctuating or lagging demand [39]. The risk of supply chain disruption is divided into demand, process, supply and environmental, and R&D investment can reduce the risk of supply chain disruption and mitigate the impact of supply chain disruption on performance from the above four dimensions, proving that R&D investment in technological innovation is one of the means to improve SCR [40]. The study finds that after the United States 9/11 incident, despite the severe economic disruption, R&D investment still has a positive impact on the performance of enterprises in the United States manufacturing and service industries [41]. This study theorizes investment—performance consecution cast in a supply chain framework, generate knowledge resources and apply knowledge through R&D investments, ultimately improving enterprise performance [42]. Based on this, Hypothesis 3 is proposed.

H3: R&D investment plays a positive and significant role in SCR and enterprise performance.

## 3. Methodology

### 3.1 Data and sample

Auto parts manufacturing enterprises are an important bridge connecting upstream and downstream in the supply chain of the automobile industry, which is one of the main profit sources of the automobile industry. Therefore, this paper selects China’s Shanghai and Shenzhen A-share automobile listed companies as the research sample. The timeframe of the study is set as 2010~2022. The data related to the study comes from CSMAR database. According to Shenyin Wanguo Industry Classification(SWIC), there are a total of 146 auto parts enterprises, in this paper, the data are processed as follows: (1) excluding the samples of enterprises with “ST” and “*ST” in the sample period; (2) excluding the samples with missing values; (3) shrinking the continuous variables above and below the 1% level, and finally obtaining 130 auto parts enterprises, 931 sample observations.

### 3.2 Variable measurement and definition

**Fig 1:**
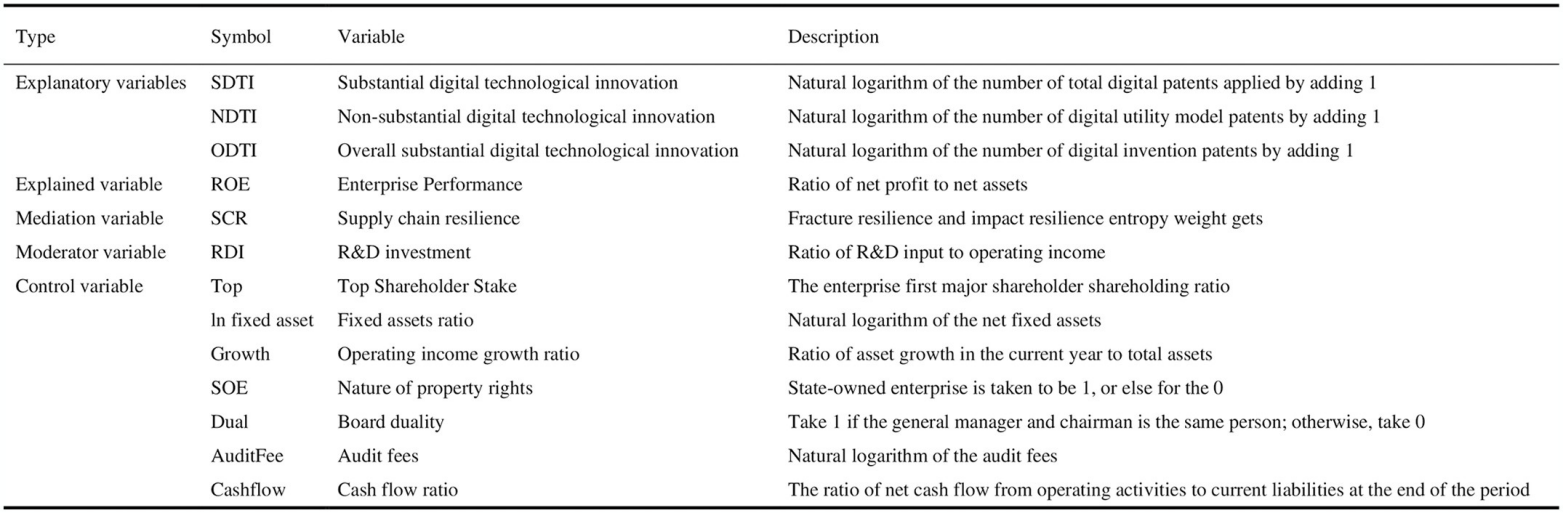
Variable description.

1. Dependent: enterprise performance(ROE), in general, the measurement of enterprise performance is divided into financial and non-financial indicators, financial indicators are usually based on the financial statements of the enterprise, and these data are usually objective and quantifiable, and can accurately respond to the economic situation of the enterprise, so this study draws on the common practice of scholars, and chooses the net assets that reflect the ratio of shareholders’ input to output (ROE) to measure enterprise performance.
2. Independent: digital technology innovation (DTI). In this paper, conduct keyword text analysis on the abstracts, descriptions and claims of all invention patents and utility model patent application documents of auto parts manufacturing enterprises, Filter out digital technology keywords. The text analysis of the content of the patent application documents was carried out to calculate the number of digital patent applications of lists companies of auto parts manufacturing enterprises in each year, and the add one to take the natural logarithm and then use the variable DTI to represent it as an indicator of enterprise DTI [43]. DTI is subdivided into three dimensions: substantial digital technological innovation(SDTI), non-substantial digital technological innovation(NDTI) and overall digital technological innovation(ODTI) [44]. we conducted natural logarithm processing and lag by one period on the patent counts.
  Supply Chain Resilience (SCR). In the field of economics, the measurement of resilience mainly has a single indicator method and a comprehensive indicator method, and the single indicator method mainly takes the change of gross domestic product or employment as the core variable [45], The application of the composite index method to SCR is more complete. Therefore, the SCR of auto parts manufacturing enterprises is divided into fracture resilience and impact resilience two levels of measurement, fracture resilience includes two aspects of robustness and liquidity, of which the robustness is measured by revenues, debt to asset ratio, and total profit. The liquidity is measured by operating expenses and R&D expenses; impact resilience includes vulnerability and developmental aspects, where vulnerability is measured by current ratio, operating profit margin, inventory turnover ratio, and developmental aspects are measured by non-current asset [46]. This paper adopts the entropy weight method to measure the weights of the above nine indicators, and finally calculates the SCR index of auto parts manufacturing enterprises, The SCR indicator is shown in Table 1.
3. Moderating variables: R&D investment. The ratio of R&D investment to operating income. This paper uses the ratio of R&D input to operating income as an indicator to quantify RDI.
4. Controls: this paper on the enterprise first major shareholder shareholding ratio(Top), the logarithm of the net fixed assets(ln fixed asset), growth(Growth)that is the annual growth rate of operating income, the nature of property rights(SOE) that is a state-owned enterprise is taken to be 1, or else for the 0, the two positions in one(Dual) that is chairman of the board of directors and general manager is the same person for 1, otherwise 0, the audit fee to take the natural logarithm(Audit Fee), cash flow ratio (Cashflow) variables to control(see Fig 1).

**Table 1:** SCR indicator.

Target level	Primary indicators	Secondary indicators	Tertiary indicators	Description of indicators
SCR	Fracture resilience	Robustness	Revenues	Operating revenues
			Debt to asset ratio	Total liabilities/total assets(%)
			Total profit	Operating profit
		Liquidity	Financial flows	Operating expense
			Technological flows	R&D expenses
	Impact resilience	Vulnerability	Current ratio	Current assets/current liabilities(%)
			Operating profit margin	Operating profit/operating revenues(%)
			Inventory turnover ratio	Operating costs/average inventory(%)
		Developmental	Non-current asset	Non-current assets

### 3.3 Model

According to H1, this paper establishes the following model 1 to test the relationship between DTI and ROE.


$$\begin{array}{c} ROE=a_{0}+a_{1}DTI+a_{2}Top+a_{3}\text{ln}\;fixedasset+a_{4}Growth+\\ a_{5}Dual+a_{6}AuditFee+a_{7}Cashflow+a_{8}SOE+\sum year+\varepsilon \end{array}$$ (1)

According to H2, this paper model 2 the relationship between SCR and ROE as follows.


$$\begin{array}{c} Mediator=b_{0}+b_{1}DTI+b_{2}Top+b_{3}\text{ln}\;fixedasset+b_{4}Growth+\\ +b_{5}Dual+b_{6}AuditFee+b_{7}Cashflow+b_{8}SOE+\sum year+\varepsilon \end{array}$$ (2)

$$\begin{array}{c} ROE=c_{0}+c_{1}DTI+c_{2}Mediator+c_{3}Top+c_{4}\text{ln}\;fixedasset+c_{5}Growth+\\ c_{6}Dual+c_{7}AuditFee+c_{8}Cashflow+c_{9}SOE+\sum year+\varepsilon \end{array}$$ (3)

According to H3, the multiplication term of RDI and SCR is generated, and the moderating effect regression is carried out by substituting model (4).


$$\begin{array}{c} ROE=a_{0}+a_{1}SCR+a_{2}Top+a_{3}\text{ln}\;fixedasset+a_{4}Growth+a_{5}Dual+a_{6}AuditFee+\\ a_{7}Cashflow+a_{8}SOE+a_{9}SCR\times RDI+\sum year+\varepsilon \end{array}$$ (4)

## 4. Empirical analysis

### 4.1 Descriptive statistics

According to the results reported in Table 2, it can be seen that: (1) the maximum value of ROE is 3, and the minimum value is -3.13, which indicates that some of the Shanghai and Shenzhen A-share listed enterprises in the automotive parts manufacturing industry have suffered losses, and the average value is 0.82, which indicates that operational state of affairs is better; (2) the minimum value of SCR is 0.44, and the maximum value is 0.66, which indicates that there is not a big gap in the level of SCR among these enterprises. The overall SCR level of auto parts manufacturing enterprises is low. (3) The minimum value of SDTI is 0, and the maximum value is 5.08, the minimum value of NDTI is 0, and the maximum value is 5, the minimum value of ODTI is 0, and the maximum value is 4.89, indicating that the DTI level of auto parts enterprises as a whole shows a large difference, and the SDTI mean value is 0.72, NDTI mean value is 0.48, ODTI mean value is 0.46, indicating that the DTI level is lows.(4) Growth, Cashflow, RDI data overall fluctuation is small, indicating that the growth rate of auto parts listed companies, cash flow ratio, R&D investment is more equal.(5) Top, ln fixed asset, Audit Fee data overall fluctuation is larger, indicating that the gap between the audit fee of auto parts listed companies, the proportion of shares held by the first largest shareholder of enterprises is more obvious.

**Table 2:** Descriptive statistics.

Variable	N	Mean	SD	Min	Max
ROA	931	0.140	0.120	-0.130	0.770
ROE	931	0.820	0.790	-3.130	3
SCR	931	0.560	0.0400	0.440	0.660
SDTI	931	0.720	1	0	5.080
NDTI	931	0.480	0.820	0	5
ODTI	931	0.460	0.800	0	4.890
RDI	931	0.0400	0.0200	0	0.290
Top	931	36.48	15.12	7.310	75.91
lnfixedasset	931	20.45	0.980	18.37	23.84
Growth	931	0.160	0.270	-0.390	1.640
SOE	931	0.210	0.410	0	1
Dual	931	0.330	0.470	0	1
AuditFee	931	13.49	1.730	0	15.99
Cashflow	931	0.0600	0.0500	-0.0800	0.180

### 4.2 Basic model

The results of regression are presented in Table 3, reports the regression results of the benchmark model of DTI to ROE, and after the introduction of control variables, SDTI, NDTI, and ODTI all show statistically significant positive coefficients, indicating that there is a strong causal relationship between DTI and ROE of auto parts manufacturing enterprises. Therefore, supporting H1.DTI provides a broader space and resources for enterprises to launch more competitive products and services, and this innovation is an important driving force for the sustainable development of enterprises, which is conducive to the enhancement of ROE. Finally, Columns (7) and (8) in Table 3 are the results of the baseline regression of the hypothesis of the relationship between SCR and ROE, from the regression results, it can be seen that the coefficient of SCR is positive and has passed the test of 1% significant level, which indicates that SCR is conducive to the improvement of ROE, and SCR can ensure that the auto parts manufacturing industry can establish a strong supply chain network and cooperative relationship in the face of external shocks and uncertainties. Avoid production delays and losses due to supply chain disruptions. This stability helps to improve the productivity and product quality of the company, which in turn enhances the performance of enterprise.

**Table 3:** Regression results of basic relationship.

	(1)	(2)	(3)	(4)	(5)	(6)	(7)	(8)
	ROE	ROE	ROE	ROE	ROE	ROE	ROE	ROE
SDTI	0.072***			0.075***				
	(0.028)			(0.025)				
NDTI		0.078**			0.085***			
		(0.034)			(0.029)			
ODTI			0.093***			0.095***		
			(0.035)			(0.031)		
SCR							4.467***	4.828***
							(0.815)	(0.947)
Top				0.003**	0.003**	0.003**		0.003*
				(0.001)	(0.001)	(0.001)		(0.001)
lnfixedasset				0.006	0.009	0.004		0.108***
				(0.026)	(0.026)	(0.026)		(0.030)
Growth				0.721***	0.723***	0.718***		0.761***
				(0.114)	(0.113)	(0.114)		(0.113)
SOE				0.020	0.030	0.013		0.059
				(0.067)	(0.067)	(0.067)		(0.066)
Dual				-0.091*	-0.096*	-0.090*		-0.056
				(0.050)	(0.051)	(0.050)		(0.049)
AuditFee				-0.012	-0.012	-0.011		-0.004
				(0.011)	(0.011)	(0.011)		(0.011)
Cashflow				6.704***	6.713***	6.692***		5.631***
				(0.570)	(0.569)	(0.571)		(0.569)
_cons	0.764***	0.778***	0.773***	0.208	0.160	0.244	-1.693***	-4.592***
	(0.034)	(0.031)	(0.031)	(0.540)	(0.536)	(0.545)	(0.469)	(1.025)
*N*	931	931	931	931	931	931	931	931
Adjusted R^2^	0.074	0.072	0.075	0.274	0.273	0.274	0.111	0.301
year	YES	YES	YES	YES	YES	YES	YES	YES

Standard errors in parentheses.

* *p* < 0.1,

** *p* < 0.05,

*** *p* < 0.01.

### 4.3 Mediation effect test

**Fig 2:**
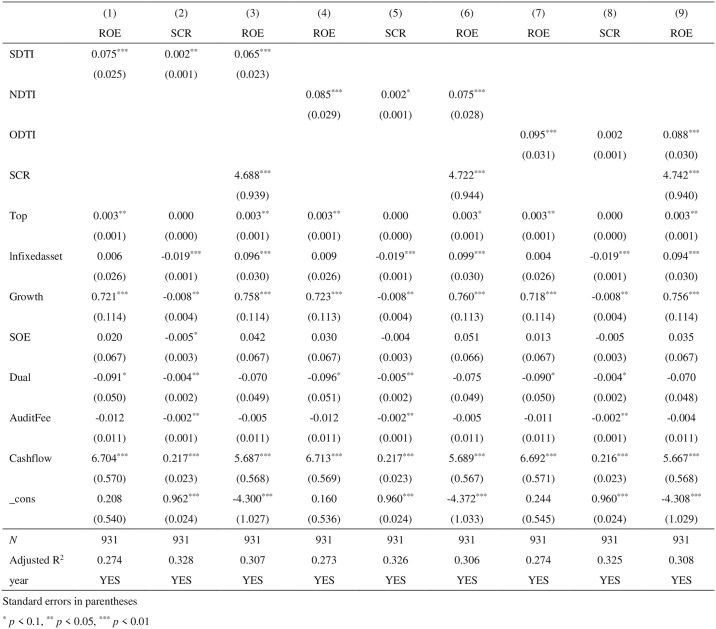
Mediation effect test.

This paper further substitutes SCR as a mediating variable into models (2) and (3) to empirically test the mediating effect of DTI on ROE, and the results are shown in Fig 2, where column (1), (4), and (7) is the basic regression result, column (2), (5), and (8) is the effect of DTI (SDTI, NDTI, and ODTI)on the mediating SCR, and column (3), (6), and (9) indicates the effect of DTI (SDTI, NDTI and ODTI) and SCR on ROE. The regression results show that column (2) SDTI passed the test of 5% significant level, indicating that SDTI has a significant role in promoting SCR; column (3) the regression coefficients of SDTI and SCR are both significantly positive, indicating that DTI can indirectly improve ROE by enhancing SCR. Although column (8) ODTI was not significant, both SDTI and NDTI showed statistically significant positive coefficients, indicating SCR as a mediating effect is established, therefore, hypothesis H2 is established.

### 4.4 Moderating effect test

Table 4 shows the regression results of model (4) to test the moderating effect of RDI between SCR and ROE. The results show that the coefficient of the cross term (128.069) is large, and the P value is less than 0.01, and the results show that the larger the RDI, the more conducive to the improvement of SCR and enterprise performance. Therefore, hypothesis 3 is validated.

**Table 4:** Moderating effect test.

	(1)
	ROE
SCR	4.982***
	(0.900)
RDI	-4.361***
	(1.173)
SCR*RDI	128.069***
	(29.586)
Top	0.003*
	(0.001)
lnfixedasset	0.102***
	(0.028)
Growth	0.747***
	(0.110)
SOE	0.056
	(0.063)
Dual	-0.042
	(0.047)
AuditFee	-0.004
	(0.010)
Cashflow	5.220***
	(0.531)
_cons	-4.353***
	(0.987)
*N*	931
Adjusted R^2^	0.333
year	YES

Standard errors in parentheses.

* *p* < 0.1,

** *p* < 0.05,

*** *p* < 0.01.

### 4.5 Robustness test

To show the completeness and reliability of the empirical analysis, the paper performs the following robustness test:

1. Alternative test of enterprise performance. This paper replaces the measure and replaces ROE with return on assets (ROA) for the test. As can be seen from the regression results of Table 5, after replacing the measure of enterprise performance, the results are consistent with the conclusions of the previous analysis.
2. Endogeneity test. ROE may be affected by DTI, and this reverse causality may lead to inconsistency and bias in our results, to further exclude the impact of endogeneity on the research results. Government subsidies are an important means for the government to directly compensate and encourage enterprises to carry out activities conducive to social development, and this paper draws on Liu et al. (2022) to explore the impact of tax reforms in government subsidies (ln gs) as an instrument [47], government subsidies are usually provided to encourage enterprise innovation and promote enterprise development. First, we conducted the first stage regression with SDTI, NDTI, and ODTI as the dependent, columns (1), (3), and (5) in Table 6 are the results of the first stage of regression, columns (2), (4), and (6) in Table 6 show the results of the second stage of regression, and the coefficient of (ln gs) passed the significant test at the 1% level, indicating that our instrumental variables are valid. The Cragg-Donald Wald F-statistic is much greater than the Stock-Yogo critical judgment value at the 10% level, which passes the weak instrument test. This indicates that after considering the endogeneity problem, DTI still has a significant contribution to the growth of ROE, and the basic regression results in this paper are reliable.

**Table 5:** Regression results of the replacing variable.

	(1)	(2)	(3)	(4)
	ROA	ROA	ROA	ROA
SDTI	0.007**			
	(0.003)			
NDTI		0.008**		
		(0.004)		
ODTI			0.010**	
			(0.004)	
SCR				1.249***
				(0.125)
Top	0.000*	0.000*	0.000**	0.000*
	(0.000)	(0.000)	(0.000)	(0.000)
lnfixedasset	-0.021***	-0.021***	-0.021***	0.004
	(0.004)	(0.004)	(0.004)	(0.004)
Growth	0.041***	0.041***	0.041***	0.051***
	(0.014)	(0.014)	(0.014)	(0.014)
SOE	-0.033***	-0.032***	-0.034***	-0.026***
	(0.008)	(0.008)	(0.008)	(0.008)
Dual	-0.006	-0.007	-0.006	0.000
	(0.008)	(0.008)	(0.007)	(0.007)
AuditFee	-0.002	-0.002	-0.002	0.000
	(0.003)	(0.003)	(0.003)	(0.003)
Cashflow	0.852***	0.853***	0.851***	0.580***
	(0.076)	(0.076)	(0.077)	(0.079)
_cons	0.524***	0.520***	0.529***	-0.688***
	(0.080)	(0.080)	(0.081)	(0.135)
*N*	931	931	931	931
Adjusted R^2^	0.262	0.262	0.263	0.362
year	YES	YES	YES	YES

Standard errors in parentheses.

* *p* < 0.1,

** *p* < 0.05,

*** *p* < 0.01.

**Table 6:** Endogeneity test.

	(1)	(2)	(3)	(4)	(5)	(6)
	SDTI	ROE	NDTI	ROE	ODTI	ROE
SDTI		0.370***				
		(0.141)				
NDTI				0.532**		
				(0.211)		
ODTI						0.481***
						(0.185)
lngs	0.173***		0.123***		0.136***	
	(0.040)		(0.033)		(0.032)	
Top	-0.001	0.005***	0.001	0.004**	-0.002	0.006***
	(0.002)	(0.002)	(0.002)	(0.002)	(0.002)	(0.002)
lnfixedasset	0.024	-0.064*	0.003	-0.056	0.036	-0.074*
	(0.045)	(0.036)	(0.037)	(0.035)	(0.036)	(0.038)
Growth	0.050	0.879***	0.019	0.884***	0.070	0.868***
	(0.123)	(0.091)	(0.102)	(0.095)	(0.098)	(0.092)
SOE	0.234***	0.053	0.089	0.088	0.257***	0.016
	(0.082)	(0.068)	(0.068)	(0.066)	(0.065)	(0.074)
Dual	0.234***	-0.167***	0.267***	-0.220***	0.176***	-0.165***
	(0.070)	(0.062)	(0.058)	(0.076)	(0.055)	(0.062)
AuditFee	-0.004	-0.047***	0.000	-0.046***	-0.008	-0.046***
	(0.021)	(0.015)	(0.017)	(0.016)	(0.017)	(0.015)
Cashflow	-0.483	6.624***	-0.525	6.710***	-0.258	6.551***
	(0.689)	(0.540)	(0.571)	(0.563)	(0.548)	(0.541)
_cons	-2.609***	1.824***	-1.708***	1.712**	-2.429***	2.056***
	(0.695)	(0.678)	(0.576)	(0.677)	(0.553)	(0.739)
*N*	931	931	931	931	931	931
Adjusted R^2^	0.082	0.071	0.059	0.000	0.090	0.062
year	YES	YES	YES	YES	YES	YES

Standard errors in parentheses.

* *p* < 0.1,

** *p* < 0.05,

*** *p* < 0.01.

### 4.6 Heterogeneity test

1. **Enterprise size** The relationship between SCR and ROE may be affected by differences in enterprise size. In this paper, the sample data are categorized according to the mean value of total assets into large enterprises(above the sample mean), small and medium-sized enterprises (below the sample mean), large enterprise group (*a*_1_ = 3.984, *p* = 0.008), small and medium-sized enterprises group (*a*_1_ = 5.568, *p* = 0.000), indicating that the regression results of both large enterprises and small and medium-sized enterprises are significant, but the coefficient of small and medium-sized enterprises is larger. Therefore, this paper concludes that the impact of SCR in terms of its effect on ROE will be greater for small and medium-sized enterprises.
2. **Nature of ownership** The relationship between SCR and ROE may be affected by enterprise ownership. In this paper, the sample data are divided into state-owned enterprises and non-state-owned enterprises for group regression. According to the regression results, state-owned enterprises (*a*_1_ = 1.199, *p* = 0.598), non-state-owned enterprises (*a*_1_ = 5.355, *p* = 0.000). The regression results of non-state-owned enterprises are all significant, and the regression results of state-owned enterprises are not significant. Therefore, the impact of SCR on ROE is significantly different between different groups of enterprise ownership (see Table 7).
3. **Region** In April 2022, the Central Committee of the Communist Party of China and the State Council issued the "Opinions of the Central Committee of the Communist Party of China and the State Council on Accelerating the Construction of a National Unified Market", In the context of the national unified market, this paper classifies China’s auto parts manufacturing enterprises registered in Jiangsu Province, Zhejiang Province, Guangdong Province, Beijing Municipality and Shanghai as enterprises in economically developed areas, and the provinces where the rest of the enterprises are located are classified as enterprises in economically less developed areas. Columns (1), (3) and (5) are the regression results of enterprises in more economically developed regions, and columns (2), (4) and (6) are the regression results of enterprises in less economically developed regions (see Table 8). According to the regression results, it can be found that the mechanism of DTI on enterprise performance is greater in the less developed regions (0.1999>0.075, 0.186>0.072, 0.279>0.103). This shows that for enterprises in less economically developed regions, DTI can significantly improve the profitability of enterprises. This will help speed up the resolution of the problem of unbalanced and inadequate economic development and promote the building of a unified national market [48].

**Table 7:** SCR heterogeneity test.

	(1)	(2)	(3)	(4)
	ROE	ROE	ROE	ROE
SCR	3.984***	5.568***	1.199	5.355***
	(1.494)	(1.214)	(2.273)	(1.073)
Top	0.003	0.004**	-0.003	0.005***
	(0.002)	(0.002)	(0.004)	(0.001)
lnfixedasset	0.115*	-0.037	0.186***	0.055
	(0.062)	(0.045)	(0.068)	(0.035)
Growth	0.446***	1.121***	0.631***	0.847***
	(0.133)	(0.182)	(0.232)	(0.129)
SOE	0.073	-0.055	-	-
	(0.089)	(0.105)	-	-
Dual	-0.136*	-0.049	-0.136	-0.108**
	(0.079)	(0.060)	(0.243)	(0.050)
AuditFee	0.001	-0.027**	0.019	-0.011
	(0.023)	(0.012)	(0.026)	(0.010)
Cashflow	5.279***	5.774***	9.083***	4.296***
	(0.954)	(0.640)	(1.693)	(0.562)
_cons	-4.199**	-1.977	-4.335**	-3.726***
	(1.873)	(1.367)	(2.136)	(1.189)
*N*	428	503	199	732
Adjusted R^2^	0.289	0.364	0.351	0.318
year	YES	YES	YES	YES

Standard errors in parentheses.

* *p* < 0.1,

** *p* < 0.05,

*** *p* < 0.01.

**Table 8:** DTI heterogeneity test.

	(1)	(2)	(3)	(4)	(5)	(6)
	ROE	ROE	ROE	ROE	ROE	ROE
SDTI	0.075**	0.199***				
	(0.034)	(0.046)				
NDTI			0.072*	0.186***		
			(0.039)	(0.055)		
ODTI					0.103**	0.279***
					(0.044)	(0.056)
Top	0.007***	0.006***	0.007***	0.006**	0.007***	0.006***
	(0.002)	(0.002)	(0.002)	(0.002)	(0.002)	(0.002)
lnfixedasset	-0.016	-0.022	-0.012	0.001	-0.018	-0.042
	(0.035)	(0.048)	(0.034)	(0.046)	(0.035)	(0.049)
Growth	0.672***	0.943***	0.669***	0.936***	0.669***	0.918***
	(0.143)	(0.199)	(0.143)	(0.202)	(0.144)	(0.197)
Dual	-0.146**	-0.168**	-0.147**	-0.181**	-0.149**	-0.168**
	(0.064)	(0.079)	(0.065)	(0.082)	(0.064)	(0.079)
AuditFee	-0.015	-0.011	-0.017	-0.012	-0.014	-0.007
	(0.021)	(0.017)	(0.022)	(0.017)	(0.022)	(0.017)
Cashflow	6.153***	6.280***	6.190***	6.281***	6.094***	6.307***
	(0.727)	(0.908)	(0.722)	(0.927)	(0.734)	(0.898)
_cons	0.630	0.586	0.584	0.175	0.663	0.962
	(0.730)	(0.976)	(0.728)	(0.951)	(0.735)	(0.999)
N	498	285	498	285	498	285
Adjusted R^2^	0.298	0.353	0.296	0.330	0.299	0.363
year	YES	YES	YES	YES	YES	YES

Standard errors in parentheses.

* *p* < 0.1,

** *p* < 0.05,

*** *p* < 0.01.

## 5. Discussion and conclusions

### 5.1 Conclusions

This paper contributes to the existing literature by studying the effects and mechanisms of DTI on enterprise performance. Our findings are consistent with existing findings [49, 50]. At the same time, our research provides a supply chain perspective, suggesting that SCR plays a mediating role in this relationship. Specifically, this paper shows that enterprise can improve their own profitability by enhancing SCR through DTI, for example, technology innovation in the Internet of Things can improve industrial competitiveness and sustainability. R&D investment plays a positive moderating role in the impact of SCR on enterprise performance, which means that adopting some joint research and development and technology sharing methods will help improve the overall stability of the supply chain.

This study emphasizes the importance of DTI to auto parts manufacturing enterprises, and the heterogeneity results show that the impact of SCR on enterprise performance is more significant among small and medium-sized enterprises, and there are significant differences among different business ownership groups. In addition, in the context of a unified market, due to the differences in China’s regional economy, DTI has a greater impact on enterprise performance in economically underdeveloped regions, which means that DTI has great value for enterprise development, it can break the restrictions of geography and economic development level, and bring new development opportunities to enterprises. It helps enterprises transform their DTI capabilities into enterprise profitability, highlighting the importance of supply chain management and DTI.

### 5.2 Theoretical implications of this study

This paper explores the mechanism between DTI and enterprise performance by providing a research perspective on supply chains, including the mediating mechanism of SCR and the mechanism of RDI moderating effect. This study contributes to the literature in two ways. First, from the perspective of supply chain, the mechanism of DTI on enterprise performance is studied through empirical analysis. Second, in different enterprise scales, business ownership and region, the role of DTI and enterprise performance mechanism in their differences is further analyzed.

Overall, this paper provides valuable theoretical contributions and some interesting reflections on the study of DTI and enterprise performance. Our findings have important implications for researchers and practitioners who are strengthening corporate profitability and improving SCR.

### 5.3 Managerial implications of this study

Based on our findings, we provide three important management takeaways that seek to improve the profitability and competitiveness of enterprise.

1. The improvement of DTI may increase R&D costs to a certain extent. Supply chain managers need to carefully assess the balance between the potential benefits of DTI and the increase in costs, and through cost-benefit analysis, select those innovation projects that can significantly improve ROE and its cost is controllable, and maintain sufficient robustness to better benefit.
2. Managers should not ignore the importance of supply chain structure for automobile parts manufacturing enterprises. The supply chain of automobile parts manufacturing enterprises usually has a multi-tier structure, which makes the supply chain more complex and increases the risk of supply chain disruption. Reasonable supply chain hierarchy design can reduce this risk and improve the transparency and predictability of the supply chain. In addition, enterprise should expand supplier diversity to reduce this dependency and build more flexible and resilient supply chains.
3. Since there is certain difference between state-owned enterprises and non-state-owned enterprises in terms of business objectives, decision-making mechanisms, and access to resources, supply chain managers need to have a deeper understanding of these difference to be able to formulate strategies and measures in a targeted manner. In view of the more urgent demand for SCR of non-state-owned enterprises, supply chain managers can strengthen cooperation with these enterprises to achieve supply chain optimization and upgrading through sharing of resources, joint research. In economically underdeveloped areas, enterprises can strengthen cooperation and jointly carry out DTI to achieve resource sharing and complementary advantages. At the same time, through technical cooperation, project cooperation, strategic alliances, etc. we can introduce digital technology and innovation achievements in developed regions to improve our own DTI capabilities and enterprise performance. Supply chain managers also need to pay continuous attention to industry development trends and market changes, and improve the efficiency and SCR through the introduction of new technologies and new means, to provide strong support for the sustainable development of enterprises.

### 5.4 Limitation and future research

Although this study provides a new research perspective for the literature on the relationship between DTI and enterprise performance, there are still some limitations that need to be addressed in future research. First, the supply chain perspective of this paper is based on a single listed auto parts listed company, and the complex supply chain network structure is not included in the model, because it is difficult to obtain enterprise network data and DTI data of non-listed companies in the empirical data, so the characteristics of supply chain networking, dynamics and virtualization in the digital era are not considered in this study. Second, based on the period from 2010 to 2022 and with the increase in the availability of data, future research can expand the scope of data, refine the research dimensions, and further enrich the mechanism research on DTI and enterprise performance. Finally, in the follow-up research, we can further explore technology transfer, technology supply development and technology trade based on cross-border supply chains from a macro perspective, not only at the regional level.

## Supporting information

S1 Data (XLSX)
